# Maternal care affects the phenotype of a rat model for schizophrenia

**DOI:** 10.3389/fnbeh.2014.00268

**Published:** 2014-08-11

**Authors:** Ruben W. M. van Vugt, Francisca Meyer, Josephus A. van Hulten, Jeroen Vernooij, Alexander R. Cools, Michel M. M. Verheij, Gerard J. M. Martens

**Affiliations:** ^1^Department of Molecular Animal Physiology, Donders Institute for Brain, Cognition and Behaviour, Centre for Neuroscience, Radboud UniversityNijmegen, Netherlands; ^2^Department of Cognitive Neuroscience, Donders Institute for Brain, Cognition and Behaviour, Radboud University Medical CentreNijmegen, Netherlands; ^3^Committee on the Neurobiology of Addictive Disorders, The Scripps Research InstituteLa Jolla, CA, USA

**Keywords:** cross-fostering, early-postnatal stress, schizophrenia, apomorphine-susceptible/apomorphine-unsusceptible rats, maternal care, apomorphine-induced gnawing, novelty-induced locomotor activity, (non-) arched-back nursing

## Abstract

Schizophrenia is a complex mental disorder caused by an interplay between genetic and environmental factors, including early postnatal stressors. To explore this issue, we use two rat lines, apomorphine-susceptible (APO-SUS) rats that display schizophrenia-relevant features and their phenotypic counterpart, apomorphine-unsusceptible (APO-UNSUS) rats. These rat lines differ not only in their gnawing response to apomorphine, but also in their behavioral response to novelty (APO-SUS: high, APO-UNSUS: low). In this study, we examined the effects of early postnatal cross-fostering on maternal care and on the phenotypes of the cross-fostered APO-SUS and APO-UNSUS animals later in life. Cross-fostered APO-UNSUS animals showed decreased body weights as pups and decreased novelty-induced locomotor activity as adults (i.e., more extreme behavior), in accordance with the less appropriate maternal care provided by APO-SUS vs. their own APO-UNSUS mothers (i.e., the APO-SUS mother displayed less non-arched-back nursing and more self-grooming, and was more away from its nest). In contrast, cross-fostered APO-SUS animals showed increased body weights as pups and reduced apomorphine-induced gnawing later in life (i.e., normalization of their extreme behavior), in line with the more appropriate maternal care provided by APO-UNSUS relative to their own APO-SUS mothers (i.e., the APO-UNSUS mother displayed more non-arched-back nursing and similar self-grooming, and was not more away). Furthermore, we found that, in addition to arched-back nursing, non-arched-back nursing was an important feature of maternal care, and that cross-fostering APO-SUS mothers, but not cross-fostering APO-UNSUS mothers, displayed increased apomorphine-induced gnawing. Thus, cross-fostering not only causes early postnatal stress shaping the phenotypes of the cross-fostered animals later in life, but also affects the phenotypes of the cross-fostering mothers.

## Introduction

Schizophrenia is a severe and complex mental illness with a neurodevelopmental origin. It affects approximately 1% of the general population (Insel, 2010), and is thought to result from an interplay between genetic and environmental factors (Sullivan et al., 2003; Tsuang et al., 2004). Previous studies have suggested that exposure to environmental risk factors during childhood, such as disrupted families and malnutrition, may lead to an increased risk of developing schizophrenia in adulthood (Galletly et al., 2011; Fryers and Brugha, 2013). Gene-environment interactions are difficult to study in humans due to the relatively large genetic variation, the timing of the events (i.e., early-life adversity determines late onset of the disease), the variability in the environment and the ethical issues involved in changing the environment (van Os et al., 2010). Animal models allow the study of such interactions in a well-controlled experimental setting at both the genetic and environmental level (Ayhan et al., 2009; Karl, 2013).

In order to investigate the interplay between genetic factors and postnatal stressors, we have used the apomorphine-susceptible (APO-SUS) rat line and their phenotypic counterpart, the apomorphine-unsusceptible (APO-UNSUS) rat line (Ellenbroek et al., 1995). APO-SUS and APO-UNSUS rats have been bred on the basis of their behavioral response to a subcutaneous (s.c.) injection of the dopamine D1/D2 receptor agonist apomorphine (Ellenbroek and Cools, 2002). With respect to this gnawing response, both APO-SUS and APO-UNSUS rats are extremes within a normal Wistar rat population; compared to Wistar rats, APO-SUS rats show a high behavioral response to apomorphine, whereas APO-UNSUS rats show a low response to apomorphine (Cools et al., 1990). APO-SUS and APO-UNSUS rats are also extremes in their locomotor response to novelty (Cools et al., 1990). Importantly, APO-SUS animals display various other schizophrenia-relevant features as well (for review: Ellenbroek and Cools, 2002), including an increased behavioral response to amphetamine (van der Elst et al., 2007), disturbed prepulse inhibition (Ellenbroek et al., 1995; Chung et al., 2011), diminished latent inhibition (Ellenbroek et al., 1995) and an increased endocrine, dopamine, and subsequent behavioral response to (environmental) challenges (Cools et al., 1990; Rots et al., 1995; van der Elst et al., 2005).

Maternal care has been shown to change behavioral and endocrine responses to stressors in various animal models (Caldji et al., 1998; Francis et al., 1999; Sequeira-Cordero et al., 2013). Ellenbroek et al. (2000) have previously shown that cross-fostering reduces apomorphine-induced gnawing in APO-SUS, but not in APO-UNSUS rats. However, cross-fostering-induced changes in the maternal care and phenotypes other than the gnawing behavior of these rats have not been investigated. Given the individual-specific behavioral responses to environmental stressors, we hypothesized that exposure of APO-SUS and APO-UNSUS mothers to pups of a different phenotype results in individual differences in maternal care. Here, we performed a detailed cross-fostering study, including a systematic analysis of the maternal care provided by APO-SUS and APO-UNSUS mothers while cross-fostering, and received by the cross-fostered pups. In addition to the effects of cross-fostering on apomorphine-induced gnawing, we also explored the effects of cross-fostering on novelty-induced locomotion. Finally, we studied whether cross-fostering alters the phenotypes of the APO-SUS and APO-UNSUS mothers.

## Materials and methods

### Animals

All experiments were performed in accordance with institutional, national and international laws and guidelines for animal care and welfare and the experiments were approved by the Radboud University Nijmegen Ethical Committee on Animal Experimentation (RU-DEC). Rats were bred in the Central Animal Laboratory of the University of Nijmegen according to methods described by Cools et al. (1990). Briefly, Wistar rats were subjected to a 1.5 mg/kg apomorphine injection (s.c.), after which they were exposed for 45 min. to a box designed to measure stereotyped gnawing (see Cools et al., 1990). This so-called “gnawing box” was slightly modified from the gnawing box described initially by Ljungberg and Ungerstedt (1978) and contains 32 holes surrounded by concentric ridges. Gnawing, on these ridges caused a characteristic vibration that was detected by a microphone, amplified and converted to digital pulses. The nine highest-gnawing animals per sex were selected to obtain the first APO-SUS generation, whereas the nine lowest-gnawing animals per sex were selected to obtain the first APO-UNSUS generation. This selection procedure was repeated for 15 generations, after which the lines were considered stable and no additional apomorphine selection tests had to be performed anymore. In this study, Nijmegen Wistar rats (APO-SUS and APO-UNSUS) of the 33rd generation of the replicate lines were used; in the previously performed cross-fostering experiment (Ellenbroek et al., 2000), the 20th generation of the original lines were used. The average gnawing score of APO-SUS animals is ~1750 gnaws in the present compared to ~800 gnaws in the previous study by Ellenbroek et al. (2000). Water and food was available *ad libitum* except during behavioral testing. The animals were housed in Makrolon type III cages (42 × 26.5 × 18 cm) with wood chip bedding located in temperature and humidity controlled rooms with a standard 12 h light/dark cycle (lights on from 07:00 h to 19:00 h).

### Breeding

One male and one female rat were placed for one night together in a special mating cage containing a wire-mesh floor. The next morning this cage was checked for a copulation plug. When the plug was found, that day was labeled embryonic day (E) 0. The pregnant females were housed in isolation and the cages were cleaned at E15 with great care to avoid stress. From E19 onwards, cages of pregnant rats were checked three times a day (at 08:00, 12:00 and 16:00 h) for the presence of pups. The day mothers gave birth to the first pup of their nest (E22 ± 0.03) was labeled postnatal day (PND)0. Nests which were born after 16:00 h were considered born at 08:00 h on the following day. Cages were again cleaned at PND14 and PND21 by only renewing the dirty bedding outside the nest. Pups were weaned at PND28, after which they were housed with one or two other rats of the same sex and nest. The breeding animals were housed together with one or two other rats of the same rat line and sex.

### Cross-fostering procedure

In the un-/cross-fostering experiment, at 16:00 h the dams were removed from their cages for a short period of time (5 min.) and were put back with their own pups (unfostered) or with pups from their phenotypic counterpart (cross-fostered) (Ellenbroek et al., 2000). At the day of weaning (PND28) the pups were weighed. The average nest sizes of APO-SUS and APO-UNSUS animals over the last four generations were checked (APO-SUS: average = 7.5 ± 0.27 pups, *n* = 113; APO-UNSUS: average = 10.8 ± 0.26 pups, *n* = 124). Extreme APO-SUS and APO-UNSUS nests (i.e., nests that varied more than two pups from their average nest size) were excluded from the experiment.

### Scoring of maternal care

To enable blind scoring of maternal behavior, the identification labels on the animals’ cages were replaced by non-descriptive codes. Maternal care scoring was performed five times a day from PND2 to PND8 during the light phase at 09:00, 12:00 and 16:00 h, and during the dark phase at 06:00 and 20:00 h for a 60-min. period, using previously described procedures (Myers et al., 1989; Champagne et al., 2003). Within an observation session, the ongoing behavior of each litter was observed every 3 min. (20 observations per session). Immediately after each of these observations, the behaviors seen were recorded on a checklist. In this way, each mother-litter combination was observed 700 times from PND2 to PND8 (7 days × five sessions per day × 20 observations per session). Red lights were used during the dark phase to enable behavioral assessment. The following behavioral features were scored (see also: Myers et al., 1989; Champagne et al., 2003): mother away from the nest (i.e., the mother is not in contact with the pups), mother licking the pups (i.e., the mothers display a licking or grooming spell directed at the pups), mother self-grooming (i.e., the mothers display a self-licking or grooming spell), non-arched-back nursing (i.e., the pups have access to the nipples, but the mother did not extend her legs, includes passive nursing and blanket nursing), arched-back nursing (i.e., the mother presents her nipples to the pups by arching over the pups).

### Behavioral studies

The cross-fostered and unfostered pups (at ~PND 60) and the cross-fostering and unfostering mothers (at ~2 weeks after weaning) were subjected to an open field test and the apomorphine susceptibility test. For the open field experiment, rats were placed in the middle of a square black table (160 × 160 cm; 95 cm elevated above the floor) surrounded by a white background illuminated by white light of 80 Lux at the middle of the open field (Verheij et al., 2008). The activity was recorded with a video camera for a period of 30 min. and analyzed with a computerized tracking system, as described by Cools et al. (1990). Two weeks after the open field test, the animals were subjected to the apomorphine susceptibility test, as described by Cools et al. (1990). The animals received an injection of 1.5 mg/kg apomorphine (s.c.) and were placed in a gnawing box (69 × 69 × 25 cm) with a central cubical (25 × 25 × 25 cm) which contained 32 holes (diameter approximately 3 cm), each of which was surrounded by five concentric ridges (Ljungberg and Ungerstedt, 1978). A microphone was placed underneath the central cubicle to allow registration and automatically scoring of the gnawing counts (Cools et al., 1990). Since the gnawing of APO-SUS rats facing one of the corners, instead of the gnawing holes, could not be automatically recorded, we excluded APO-SUS animals that spent more than 12% of the total observation time (45 min.) in the corners (Cools et al., 1990).

### Statistics

Maternal care was statistically analyzed using a two-way ANOVA with the factors rat type (APO-SUS and APO-UNSUS) and treatment (cross-fostered and unfostered), followed by *post hoc* Student’s *t*-tests where appropriate. The level of significance was set at *p* < 0.05. Given that open field locomotor activity of the pups at adulthood (*t*_(141)_ = −6.3, *p* < 0.05) and weight at PND28 (*t*_(134)_ = 2.5, *p* < 0.05) significantly differed between control males and females, the results regarding these two measures were analyzed per gender (see Figures 2,[Fig F3] 4 below). Maternal care received by the pups was analyzed in groups containing at least six mothers per group. The groups in which the measurements in the (grown-up) pups were obtained (weight, open field and apomorphine-induced gnawing score) consisted of at least 20 animals and were derived from at least four different nests. Data are expressed as average ± standard error of mean (SEM).

**Figure 1:**
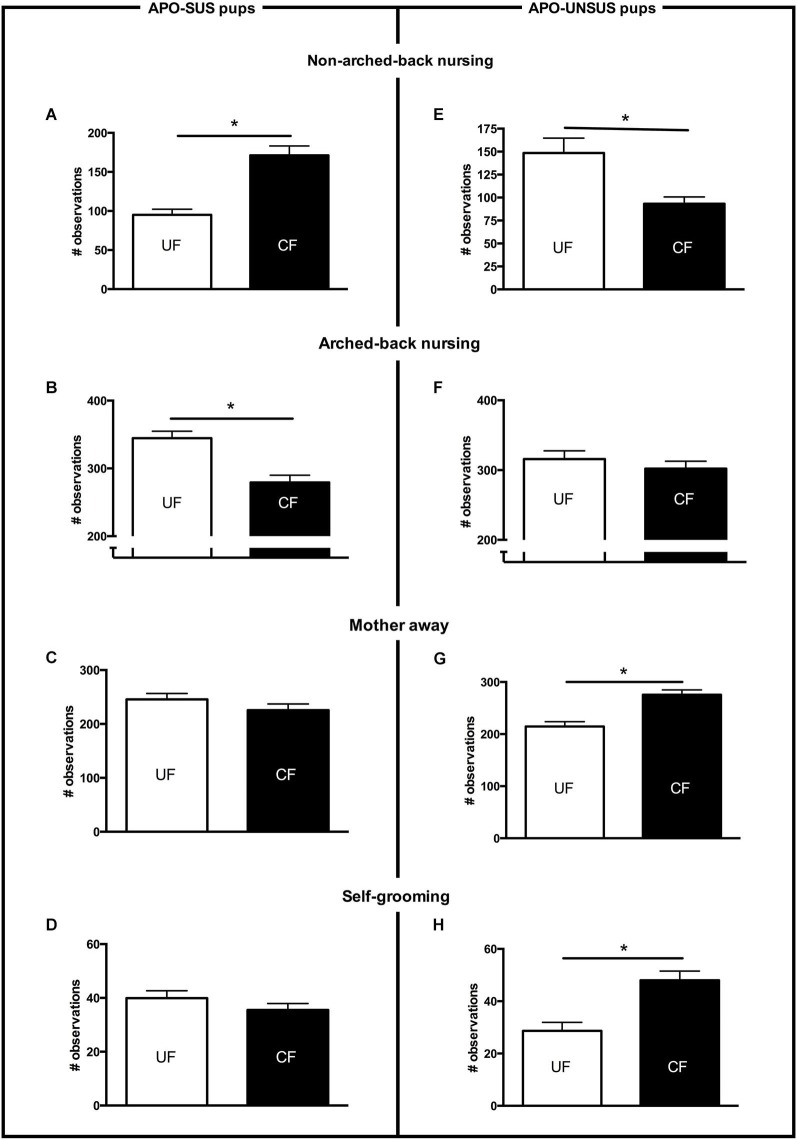
**Maternal care received from PND2 to PND8 by APO-SUS (left panel) and APO-UNSUS pups (right panel) when being unfostered (UF; white bars) or cross-fostered (CF; black bars)**. **(A)** and **(E)**: non-arched-back nursing; **(B)** and **(F)**: arched-back nursing; **(C)** and **(G)**: mother away; **(D)** and **(H)**: self-grooming. The total number of observations per nest was 700. * *p* < 0.05. Note: To avoid nest disturbance, gender-specific maternal care was not analyzed (see Section Discussion).

**Figure 2:**
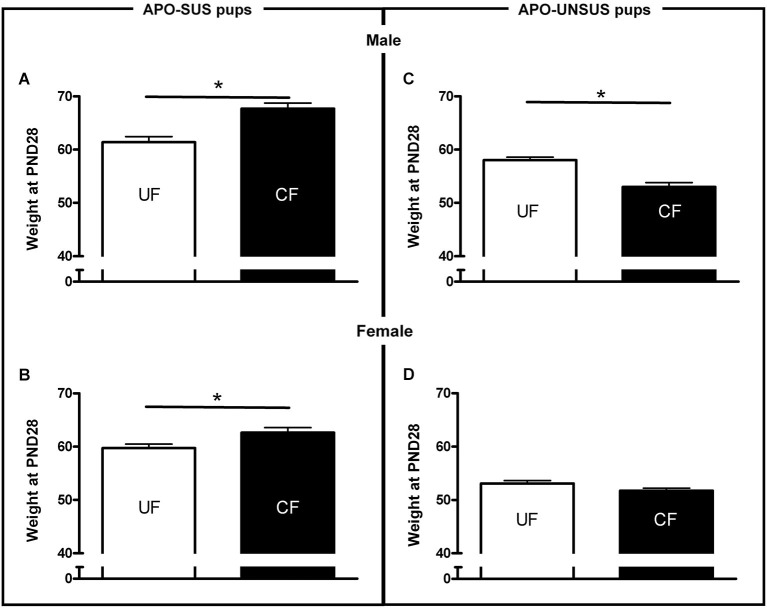
**Weights at PND 28 of male (A) and female (B) APO-SUS, as well as male (C) and female (D) APO-UNSUS pups after being unfostered (UF; white bars) or cross-fostered (CF; black bars).** * *p* < 0.05.

**Figure 3 F3:**
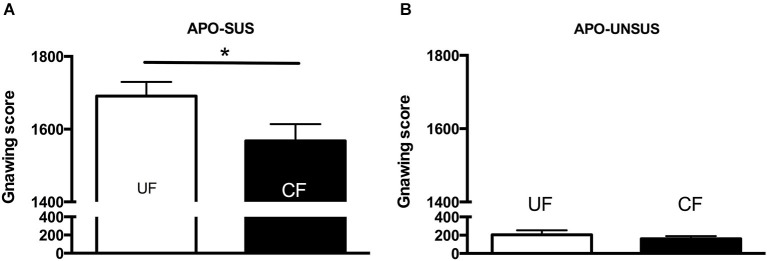
**Apomorphine-induced gnawing of APO-SUS (A) and APO-UNSUS (B) rats after being unfostered (UF; white bars) or cross-fostered (CF; black bars) in their early life.** * *p* < 0.05. Note: No gender differences were observed.

**Figure 4:**
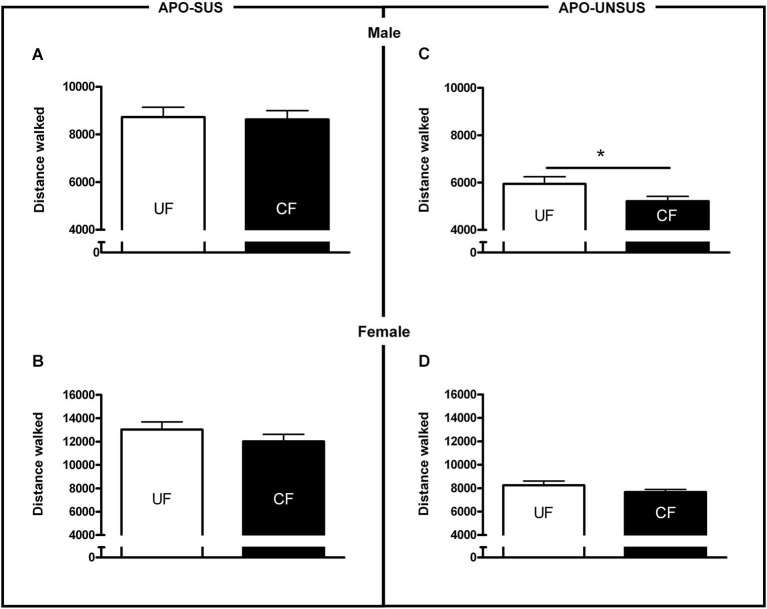
**Novelty-induced locomotion in male APO-SUS (A) and APO-UNSUS (B) pups as well as female APO-SUS (C) and APO-UNSUS (D) pups after being unfostered (UF; white bars) or cross-fostered (CF; black bars).** * *p* < 0.05.

## Results

### APO-SUS- and APO-UNSUS-specific effects of cross-fostering

APO-SUS- and APO-UNSUS-specific effects of cross-fostering were found for all measures of maternal care (except for licking of the pups). A two-way ANOVA revealed significant rat type × treatment interactions for non-arched-back nursing (*F*_(1,40)_ = 36.7, *p* < 0.05), arched-back nursing (*F*_(1,40)_ = 4.89, *p* < 0.05), the frequency of the mother away from the nest (*F*_(1,40)_ = 12.37, *p* < 0.05) and the self-grooming frequency of the mother (*F*_(1,40)_ = 12.94, *p* < 0.05). In addition, cross-fostering differentially affected the weights of the APO-SUS and APO-UNSUS pups (rat type × treatment interaction: males *F*_(1,162)_ = 33.47, *p* < 0.05, females *F*_(1,171)_ = 7.61, *p* < 0.05). Rat-type-specific effects of the cross-fostering were also found at adulthood (gnawing: *F*_(1,38)_ = 5.16, *p* < 0.05). The results of the *post hoc* analyses are described below.

### Maternal care received by the APO-SUS and APO-UNSUS pups

Cross-fostered APO-SUS pups received significantly more non-arched-back nursing (*t*_(22)_ = −5.2, *p* < 0.05, Figure 1A), which was accompanied by a similar decrease in arched-back nursing (*t*_(22)_ = 4.4, *p* < 0.05, Figure 1B). The other maternal behaviors (mother away: *t*_(22)_ = 1.2, n.s., Figure 1C; mother self-grooming: *t*_(22)_ = 1.2, n.s., Figure 1D; mother licking the pups: *t*_(22)_ = 0.5, n.s, data not shown) were not different between cross-fostered and unfostered APO-SUS nests. In contrast, cross-fostered APO-UNSUS pups received significantly less non-arched-back nursing (*t*_(18)_ = 3.6, *p* < 0.05, Figure 1E), but arched-back nursing was not significantly different (*t*_(18)_ = 0.8, n.s, Figure 1F). The APO-SUS mothers fostering APO-UNSUS pups were more frequently away from their nests (*t*_(18)_ = −3.9, *p* < 0.05, Figure 1G) and showed more frequently self-grooming (*t*_(18)_ = −3.3, *p* < 0.05, Figure 1H). Licking of the pups by the mother was not significantly different between cross-fostered and unfostered APO-UNSUS nests (*t*_(18)_ = 0.1, *p* < 0.05, data not shown).

### Weights of APO-SUS and APO-UNSUS pups at PND28

As previously shown (Degen et al., 2005), unfostered APO-SUS pups were heavier than unfostered APO-UNSUS pups (male: *t*_(67)_ = 2.7, *p* < 0.05, correlation with nest size *p* < 0.05 (Pearson’s analysis); female: *t*_(64)_ = 6.3, *p* < 0.05, correlation with nest size *p* < 0.05 (Pearson’s analysis). Cross-fostered APO-SUS pups weighed significantly more than unfostered APO-SUS pups at PND28 (male: *t*_(89)_ = 4.2, *p* < 0.05, Figure 2A; female: *t*_(87)_ = 2.5, *p* < 0.05, Figure 2C), whereas cross-fostering resulted in a significant (male-specific) reduction of body weights in APO-UNSUS pups at PND28 (male: *t*_(73)_ = −4.6, *p* < 0.05, Figure 2B; female (*t*_(84)_ = −1.5, n.s., Figure 2D).

### Behavior of unfostered/cross-fostered APO-SUS and APO-UNSUS animals at adulthood

Cross-fostered APO-SUS pups showed a decreased apomorphine-induced gnawing score at adult age compared to unfostered APO-SUS animals (*t*_(186)_ = −2.0, *p* < 0.05, Figure 3A), whereas cross-fostered APO-UNSUS pups displayed no change in apomorphine-induced gnawing at adulthood (*t*_(93)_ = −0.83, n.s., Figure 3B). A small, but significant, (male-specific) decrease of novelty-induced locomotor activity was observed in cross-fostered APO-UNSUS pups at adult age (male: *t*_(88)_ = −2.1, *p* < 0.05, Figure 4B; female: *t*_(97)_ = −1.4, n.s., Figure 4D). In contrast, cross-fostering had no effect on locomotor activity on the open field in APO-SUS rats at adulthood (male: *t*_(99)_ = −0.17, n.s., Figure 4A; female: *t*_(78)_ = −1.1, n.s., Figure 4C).

### Behavior of the APO-SUS and APO-UNSUS mothers after weaning

**Figure 5:**
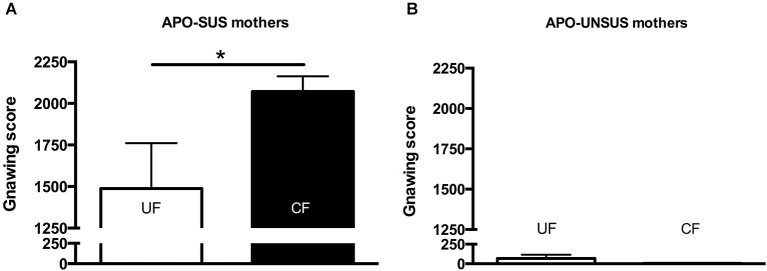
**Apomorphine-induced gnawing of APO-SUS (A) and APO-UNSUS (B) mothers after fostering their own pups (white bars) or fostering pups from their phenotypic counterpart (black bars).** * *p* < 0.05.

Two weeks after the mothers were separated from their pups, APO-SUS mothers having fostered APO-UNSUS pups showed a significant increase in apomorphine-induced gnawing when compared to APO-SUS mothers that had fostered their own pups (*t*_(21)_ = 2.4, *p* < 0.05, Figure 5A), whereas cross-fostering had no effect on the gnawing behavior of the APO-UNSUS mothers (*t*_(17)_ = 0.2, n.s., Figure 5B). The novelty-induced locomotor activity on the open field did not significantly differ between mothers having fostered their own pups and mothers having fostered pups of their phenotypic counterpart (APO-SUS mothers: *t*_(16)_ = −1.1, n.s.; APO-UNSUS mothers: *t*_(16)_ = −0.4, n.s., data not shown).

A summary of the results from our cross-fostering experiments with APO-SUS and APO-UNSUS rats is presented in Table 1.

**Table 1:** **Overview of the results from the cross-fostering experiments with APO-SUS and APO-UNSUS rats**.

	**PND2—8**	**PND28**	**Adulthood (>PND60)**	
	**Maternal care received**	**Weight**	**Open field**	**Gnawing score**
CF vs. UF APO-SUS rats	Non-arched-back-nursing ↑ Arched-back nursing ↓	↑	=	↓
CF vs. UF APO-UNSUS rats	Non-arched-back nursing ↓ Mother away ↑ Mother self-grooming ↑	↓ (males)	↓ (males)	=

*Maternal care was scored from PND2 to PND8. At weaning (PND28), the weights of the cross-fostered (CF) and unfostered (UF) pups were determined. The phenotypes of the grown-up pups were determined after PND60*.

## Discussion

In this study, we performed cross-fostering experiments to determine whether a change in the maternal care of pups may alter their later-life phenotypes and what the effects of disrupted mother-pup interactions are on the dams. An important indicator reflecting the quality of maternal care received by a pup constitutes its body weight. The body weight of a pup is known to be heavily determined by the amount of nutrients and body warmth provided by the mother (Stone et al., 1976; Smart et al., 1983). With respect to nutritional supply, it is important to note that maternal care can be provided by arched-back or non-arched-back nursing. Despite the fact that the non-arched-back position is generally considered to be less favorable for feeding than the arched-back position (Lonstein et al., 1998), we found that in cross-fostered APO-SUS pups an increase of non-arched-back nursing, together with a similar decrease of arched-back nursing, was accompanied by increased body weights. Apparently, despite this form of behavioral competition (i.e., non-arched-back nursing replaces arched-back nursing), the APO-SUS pups were still able to suckle the nipples to receive enough milk even when the APO-UNSUS mother was in a less favorable position. In addition, the more pronounced skin-to-skin warmth provided by the higher level of non-arched-back nursing of the APO-UNSUS mothers may also have contributed to the observed increased body weights of the cross-fostered APO-SUS pups. The decrease in non-arched-back nursing received by APO-UNSUS pups when they were fostered by APO-SUS mothers likely results not only in less milk received by the pups but also in less skin-to-skin warmth (Kojima and Alberts, 2011). The decreased non-arched-back nursing received by cross-fostered APO-UNSUS pups was indeed accompanied by a decrease in their body weights. These observations indicate that APO-SUS mothers fostering APO-UNSUS pups provided less maternal care than cross-fostering APO-UNSUS mothers. Cross-fostering APO-SUS mothers showed indeed more self-grooming and more time away from the pups. The lower quality of maternal care provided by cross-fostering APO-SUS vs. APO-UNSUS mothers may be explained by the fact that APO-SUS rats show a stronger and longer endocrine, neurochemical, and subsequently behavioral response to environmental challenges (i.e., in this study exposure to pups of their phenotypic counterpart) than APO-UNSUS rats (Rots et al., 1995).

Ellenbroek et al. (2000) have previously found that cross-fostering reduced apomorphine-induced gnawing in APO-SUS, but not in APO-UNSUS rats. The more pronounced decrease of cross-fostering-induced gnawing observed in the previous study may be due to the fact that here we used an APO-SUS rat line resulting from a different pharmacogenetic selection and bred for 12 more generations than the previous line. Importantly, in the present study we performed additional behavioral studies showing that cross-fostering reduces novelty-induced locomotion in APO-UNSUS but not in APO-SUS rats. The observed decrease in body weight and the more extreme phenotype (i.e., reduced locomotion) in cross-fostered APO-UNSUS rats may be attributed to the less appropriate maternal care of APO-SUS mothers (i.e., less non-arched-back nursing, more observations of the mother being away and more self-grooming). In contrast, the observed increase in body weight and the normalization of the phenotype (i.e., reduced gnawing) in cross-fostered APO-SUS rats may very well be explained by the more appropriate maternal care provided by APO-UNSUS mothers (i.e., more non-arched-back nursing, and no change in the number of observations of the mother being away or self-grooming).

In order to disturb the nests as little as possible, we performed whole-nest observations, which implied that maternal care could not be analyzed per pup. In contrast, in other studies the nests have been disturbed to examine mother-pup interactions per pup—either by labeling the pups individually (Champagne et al., 2003) or by adjusting the gender composition of the litter to obtain either single-sex (male or female) or mixed-sex (half male and half female) litters (Hao et al., 2011). Interestingly, in the latter study mothers were found to spend more time licking male than female pups, presumably explaining that maternal deprivation had opposite effects on neurodevelopment between males and females (Oomen et al., 2009). The sex-specific effects of cross-fostering on pup body weight and adult open field behavior that we observed, suggest that cross-fostering APO-SUS and APO-UNSUS mothers may also have taken care of male and female pups differently.

To our knowledge, the effects of cross-fostering on the mothers themselves have not been described before. In this study, we observed 2 weeks after weaning an increase in apomorphine-induced gnawing in APO-SUS mothers that fostered APO-UNSUS pups, whereas APO-UNSUS mothers were unaffected by cross-fostering. We hypothesize that this long-lasting increase in apomorphine-induced gnawing (reflecting a more extreme phenotype) is due to the exposure to pups of a different phenotype, resulting in a stronger and longer-lasting stress response in APO-SUS than APO-UNSUS mothers (see also Rots et al., 1995). Indeed, chronic stress has been found to increase apomorphine-induced stereotypic behavior (Cabib et al., 1984).

## Conclusions

Although most mother-pup interaction studies have focused on the role of arched-back nursing (Caldji et al., 2003; Champagne et al., 2003; Daskalakis et al., 2012), we demonstrate that non-arched-back nursing is also important for the proper growth of the pups. In addition, the more stress-sensitive APO-SUS rats (Rots et al., 1995) provide less maternal care when they foster APO-UNSUS pups, resulting in pups with reduced body weights and more extreme adult phenotypes. The finding that the body weights of APO-SUS pups increased and the adult phenotypes of these animals became less extreme after being fostered by relatively stress-resistant APO-UNSUS mothers opens the intriguing perspective for early-life environmental intervention approaches (e.g., prevent exposure to stressful events) in humans predisposed to schizophrenia. Finally, cross-fostering not only influences the quality of maternal care, but may also have a long-lasting impact on stress-sensitive mothers. This may very well affect the phenotype of the next litter of these mothers. In conclusion, the effect of cross-fostering is not only determined by the background of the pups and their mother, but also by mother-pup interactions.

## Conflict of interest statement

The authors declare that the research was conducted in the absence of any commercial or financial relationships that could be construed as a potential conflict of interest.
